# Blood Lines: Intraspecific and Interspecific Variations in Anticoagulant Actions of *Agkistrodon* Viperid Venoms

**DOI:** 10.3390/toxins16070291

**Published:** 2024-06-26

**Authors:** Francisco C. P. Coimbra, Elda E. Sanchez, Bruno Lomonte, José María Gutiérrez, Juan J. Calvete, Bryan G. Fry

**Affiliations:** 1 Adaptive Biotoxicology Lab, School of the Environment, University of Queensland, St Lucia, QLD 4072, Australia; francisco.cp.coimbra@gmail.com; 2 National Natural Toxins Research Center, Department of Chemistry, Texas A&M University-Kingsville, MSC 224, 975 West Avenue B, Kingsville, TX 78363, USA; elda.sanchez@tamuk.edu; 3 Instituto Clodomiro Picado, Facultad de Microbiología, Universidad de Costa Rica, San José 11501, Costa Rica; bruno.lomonte@ucr.ac.cr (B.L.); jose.gutierrez@ucr.ac.cr (J.M.G.); 4 Laboratorio de Venómica Evolutiva y Traslacional, Instituto de Biomedicina de Valencia, CSIC, 46010 Valencia, Spain; jcalvete@ibv.csic.es

**Keywords:** *Agkistrodon*, viper, venom, evolution, coagulation

## Abstract

This study investigated the intraspecific and interspecific variability in the venom effects of *Agkistrodon* viperid snake species and subspecies (eleven venoms total) on plasma clotting times, fibrinogen levels, and fibrin clot strength. Significant delays in plasma clotting time were observed for *A. conanti*, *A. contortrix mokasen*, *A. contortrix phaeogaster*, *A. howardgloydi*, *A. piscivorus leucostoma*, and *A. piscivorus piscivorus*. Notably, the phylogenetically disjunct lineages *A. conanti*, *A. contortrix mokasen*, and *A. howardgloydi* exhibited the most potent anticoagulant effects, indicating the independent amplification of a basal trait. Inhibition assays with the activated clotting enzymes Factors XIa, IXa, Xa, and IIa (thrombin) revealed that FXa inhibition is another basal trait amplified independently on multiple occasions within the genus, but with *A. howardgloydi*, notably more potent than all others. Phospholipid degradation and zymogen destruction were identified as mechanisms underlying the variability in venom effects observed experimentally and in previous clinical reports. Thromboelastography demonstrated that the venoms did not clot fibrinogen directly but affected fibrin clot strength by damaging fibrinogen and that thrombin was subsequently only able to cleave into weak, unstable clots. The ability to activate Protein C, an endogenous anticoagulant enzyme, varied across species, with some venoms exceeding that of *A. contortrix contortrix*, which previously yielded the protein diagnostic agent Protac^®^. Phylogenetic analysis suggested that both fibrinogen degradation and Protein C activation were each amplified multiple times within the genus, albeit with negative correlation between these two modes of action. This study highlights the evolutionary, clinical, and biodiscovery implications of venom variability in the *Agkistrodon* species, underscoring their dynamic evolution, emphasising the need for tailored clinical approaches, and highlighting the potential for novel diagnostic and therapeutic developments inspired by the unique properties of snake venoms.

## 1. Introduction

The genus *Agkistrodon* within the Viperidae snake family evolved from Asian pit vipers that crossed the Bering Strait. These snakes are found in North America, having adapted to a wide range of habitats from wetlands to dry forests and ranging from the southeastern United States down to the northern province of Guanacaste in Costa Rica [1]. The *Agkistrodon* genus is divided into two clades; the copperhead clade, consisting of *A. contortrix contortrix*, *A. contortrix mokasen*, *A. contortrix phaeogaster*, *A. laticinctus pictigaster*, and *A. laticinctus laticinctus*, are specialists of wooded areas and rocky terrains, while the moccasin clade, consisting of *A. bilineatus*, *A. conanti*, *A. howardgloydi*, *A. piscivorus leucostoma*, *A. piscivorus piscivorus*, and *A. taylori*, are specialists to aquatic environments like swamps, streams, and marshes, with the exception of the southernmost species, *A. howardgloydi*, which has secondarily adapted to tropical dry forests [2]. *Agkistrodon* venoms have been shown to be dominant by snake venom metalloproteases (SVMPs), Group II phospholipase A_2_ (PLA_2_), and kallikrein-type serine proteases, with conserved proteolytic and phospholipase activities but differential haemorrhagic and myotoxic activities [3]. *Agkistrodon* envenomations may produce local and systemic effects; local symptoms include swelling, severe pain, bruising, blistering, and potentially significant tissue damage around the bite area, while the systemic signs and symptoms in severe cases include bleeding disorders, shock, or even organ failure, particularly if the venom components interfere with blood clotting or cardiovascular functions [4,5,6,7,8,9,10]. Most reports, however, are on *A. c. contortrix*, with few on other *A. contortrix* subspecies and scant reports for other species.

Snake venoms have long been studied for their complex biochemical properties and are primarily made up enzymatic and non-enzymatic proteins evolutionarily honed to alter any physiological pathway reachable by the bloodstream [11]. They represent an abundant source of bioactive molecules with a yet unrealised scope of potential medical applications in the leading areas of human death and disability, such as cardiovascular disease, diabetes, cancer, and chronic pain [12]. The coagulotoxic effects of snake venoms, while being critical for prey immobilisation and therefore predatory success and snakes’ survival, are of particular significant interest in medical research for potential therapeutic applications, especially in the treatment of conditions related to thrombosis and cardiovascular disease. A myriad of toxin types have been isolated and characterised from snake venoms that disrupt blood coagulation, with the venoms of viperid snakes being particularly rich in such toxins. The effects of anticoagulant toxins include the inhibition of the clotting enzymes thrombin, VIIa, Xa, IXa, XIa, and XIIa and the depletion of fibrinogen levels, leading to death from haemorrhagic shock or internal bleeding [13,14,15,16,17,18,19,20,21,22].

Platelet-mediated primary clotting is disrupted by the binding of venom toxins to a variety of platelet receptors or destruction of platelet cofactors such as the von Willebrand factor (vWF). In primary haemostasis, platelets are activated and alter their shape in a way that presents the fibrinogen binding receptor, thus facilitating platelet cross-linking and aggregation. The main families of toxins that interfere with platelet aggregation are 3FTxs (three-finger toxins) [23], L-amino acid oxidases [24], the lectin framework (including classical lectins and derived SNACLECS lectin-like proteins) [25], regions of the prepro region of the natriuretic peptide precursor [26], Group I PLA_2_ proteins [27], Group II PLA_2_ proteins [28], and the snake venom metalloproteinase (SVMP) disintegrin domain, which is released from P-II SVMPs by post-translational proteolytic cleavage and expressed as a monodomain in extremely derived forms [29].

The few direct inhibitors of the secondary clotting’s extrinsic and intrinsic pathways that have been characterised from snake venom include a dimeric 3FTx complex from *Hemachatus haemachatus* that inhibits FVIIa [30]; a monomeric 3FTx, also from *H. haemachatus*, that inhibits the extrinsic pathway activation of FX [31]; and a Kunitz peptide from *Bungarus fasciatus* that inhibits FXIa [32]. FIXa has been shown to be inhibited by lectin-like toxins from viperid snake venoms [25,33].

In contrast to the paucity of described inhibitors of the extrinsic or intrinsic pathways, a myriad of snake venom enzymes have been described that promote coagulopathic effects upon blood coagulation through deleterious actions upon the common pathway, which may contribute to the characteristic haemorrhagic syndrome in snakebite envenomings. The activation of Protein C by kallikrein-type serine proteases would inhibit FVa and FVIIIa upstream and consequently inhibit FX activation and prothrombinase formation indirectly downstream [34,35,36,37,38]. Direct inhibition of FXa has been shown for Group I PLA_2_ from *Naja* (cobra) venoms [27,39,40,41] in addition to lectin toxins from viperid snake venoms [25,33]. Lectin toxins that are inhibitors of prothrombin or thrombin (or both) have been isolated and characterised [25,42,43,44,45,46,47].

A major target for anticoagulant venoms acting upon the common pathway is fibrinogen depletion, either through destructive cleavage of fibrinogen, or cleavage in a peudo-procoagulant (aka: thrombin-like) manner such that the fibrin clots that are directly formed by the venom are weak and transient, thereby quickly breaking down [38,48]. The latter are pseudo-procoagulant in that the clots are transient and result in a net outcome of an anticoagulative state. We speculate this to be a consequence of a compromised capacity of the venom-cleaved fibrinogen chains to correctly polymerise into stable, interlinked fibrin meshes. These actions should not be confused with true procoagulant actions in which the cleavage of fibrinogen into fibrin retains its full polymerisation capabilities and functional potential due to the venom activating an upstream clotting enzyme, such as converting Factor VII into FVIIa, Factor X into FXa, or Factor II (prothrombin) into FIIa (thrombin) [16,21,48,49,50,51,52]. Both non-clotting and pseudo-procoagulant (transient clotting) cleavage of fibrinogen lead to a depletion of the levels of normal and intact fibrinogen available for clotting. In addition, the fibrinogen degradation products may also be bound by thrombin and thus also reduce the amount of thrombin available for clot formation [29,38]. Both outcomes would directly contribute to the haemorrhagic effects of these kinds of snake venoms.

Fibrinogenolysis in a non-clotting manner has been described for kallikrein-type serine proteases in addition to metalloproteases (SVMPs) in snake venoms [29,38]. Kallikrein-type enzymes may degrade both the Aα- and Bβ-chains or display selectivity for the Aα-chain or Bβ-chain [38]. Consistent with the single early origin of the Toxicofera venom system, the kallikrein-type serine proteases shared between the venoms of anguimorph lizards and those of snakes share this plesiotypic activity [38,53]. The plesiotypic (basal) state is the ability to degrade both chains, and the specialisation of one or the other chain is an apotypic (derived) activity. Degrading both the Aα- and Bβ-chains is also the plesiotypic state of P-III SVMP, but chain-specific versions have been described [29,54]. Thus, venom-induced fibrinogenolysis is a consequence of the action of pseudo-procoagulant enzymes, which form feeble fibrin clots that are readily degraded, or the action of enzymes that degrade fibrinogen and render it unclottable.

In addition to fibrinogenolysis, some snake venom enzymes have been shown to directly lyse fibrin clots [38,55]. Indirect clot lysis is also accomplished by kallikrein-type serine proteases activating plasminogen into plasmin [38,56,57,58,59]. Unlike plasminogen activation by bacteria [60,61,62,63], the snake venom activation of plasminogen occurs via proteolytic cleavage of the zymogen into the active enzymatic form. Other mechanisms of snake venoms for increasing the amount of available plasmin in order to destroy any clots formed in response to damage to the vascular bed are mediated by kallikrein-type serine proteases and metalloproteases that cause the release of the endogenous plasminogen activator from endothelial cells [29,38].

Despite its evolutionary novelty and medical importance, there is a notable deficiency in studies examining intra-species and inter-species variability in venom coagulotoxicity within the *Agkistrodon* genus [3], with questions remaining as to the contribution of such diversity to the variability in reported clinical coagulotoxic effects [4,64]. This incomplete analysis limits our understanding of the venom’s evolutionary history, potential clinical effects, and biomedical potential. Addressing these gaps through targeted research is critical not only for advancing our fundamental scientific knowledge but also for harnessing *Agkistrodon* venoms’ therapeutic utility.

## 2. Results and Discussion

**Figure 1:**
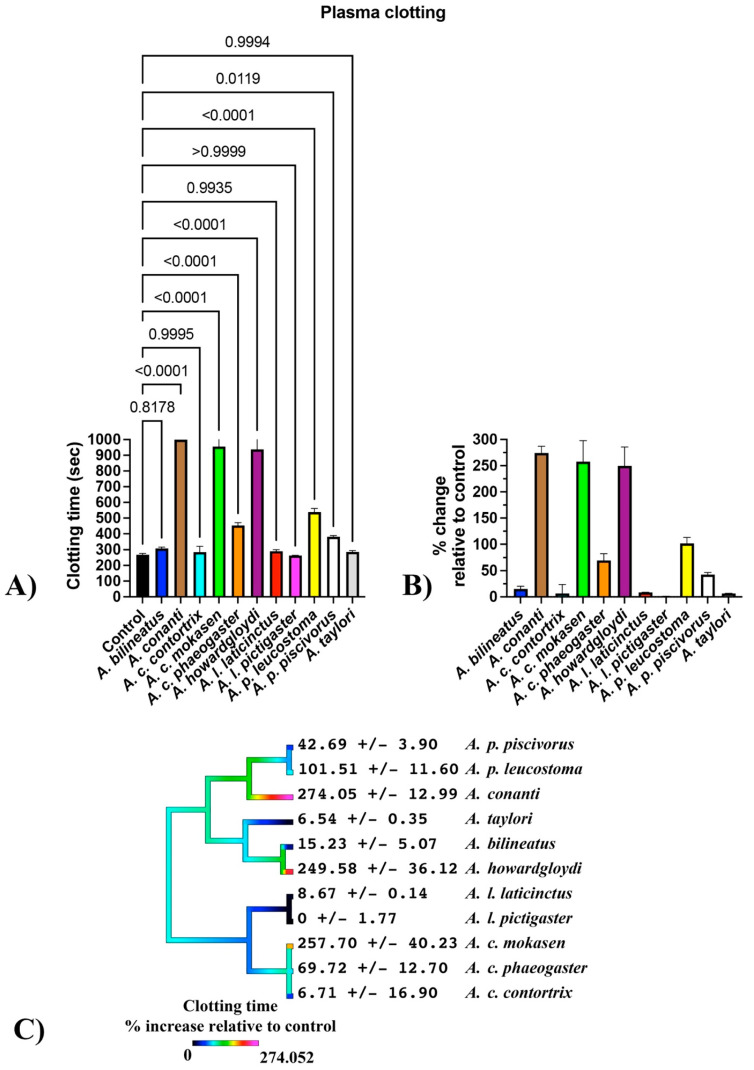
Venom impacts upon clotting of human plasma. (**A**) Raw time numbers (machine maximum: 999 s). *p*-values for each venom relative to control are from Brown–Forsythe and Welch ANOVA tests with post hoc Dunnett T3 multiple comparisons. (**B**) Proportional increases in clotting time relative to the control (no venom effect = 0%). (**C**) Ancestral reconstruction of the relative effects upon clotting time; phylogeny based upon Burbrink [2]. Data are n = 4 mean ± standard deviation.

Plasma clotting time was significantly delayed for *A. conanti*, *A. contortrix mokasen*, *A. contortrix phaeogaster*, *A. howardgloydi*, *A. piscivorus leucostoma*, and *A. piscivorus piscivorus*, with no clotting within a machine maximum time of 999 seconds for all four *A. conanti* replicates and all but one replicate for *A. contortrix mokasen* and *A. howardgloydi* (Figure 1A), with significant shifts relative to the control (Figure 1B). As the three most potent venoms (*A. conanti*, *A. contortrix mokasen*, and *A. howardgloydi*) do not represent a monophyletic clade (Figure 1C), this suggests that independent amplification of net anticoagulation potency on clotting time has occurred on at least three separate occasions within the genus (Figure 1). This represents convergent evolution, suggesting selection forces geared towards the same functional outcome. Subsequent tests to ascertain sites of action included incubation with activated enzymes or incubation with plasma followed by the addition of a specific enzyme to determine if downstream zymogens or cofactors were affected.

**Figure 2:**
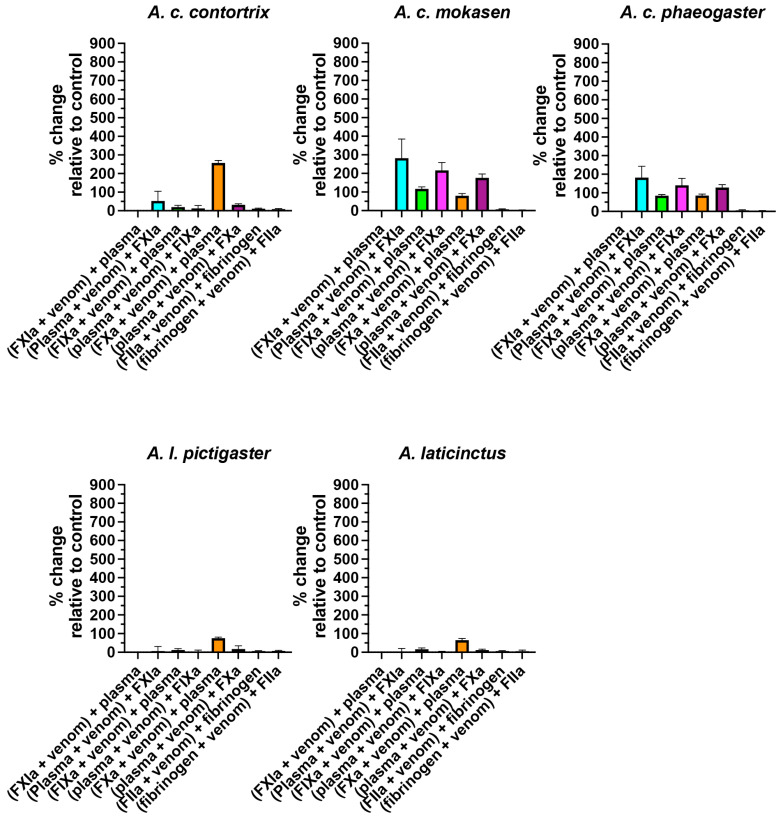
Copperhead clade proportional increase in clotting time relative to the control for each test (no venom effect = 0%). Note: to allow for comparison across venoms, all graphs are scaled relative to the point of greatest impact, which is (plasma + venom) + FIXa for *A. conanti* in Figure 3. Data are n = 4 mean ± standard deviation.

**Figure 3:**
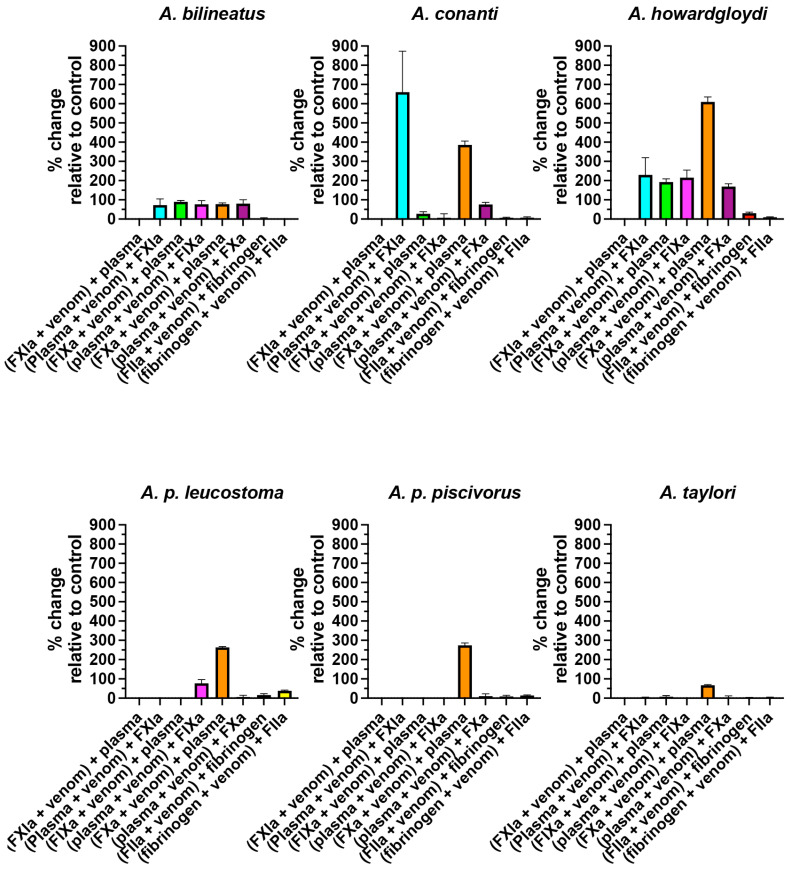
Moccasin clade proportional increase in clotting time relative to the control for each test (no venom effect = 0%). Note: to allow for comparison across venoms, all graphs are scaled relative to the point of greatest impact, which is (plasma + venom) + FIXa for *A. conanti* in this figure. Data are n = 4 mean ± standard deviation.

**Figure 4:**
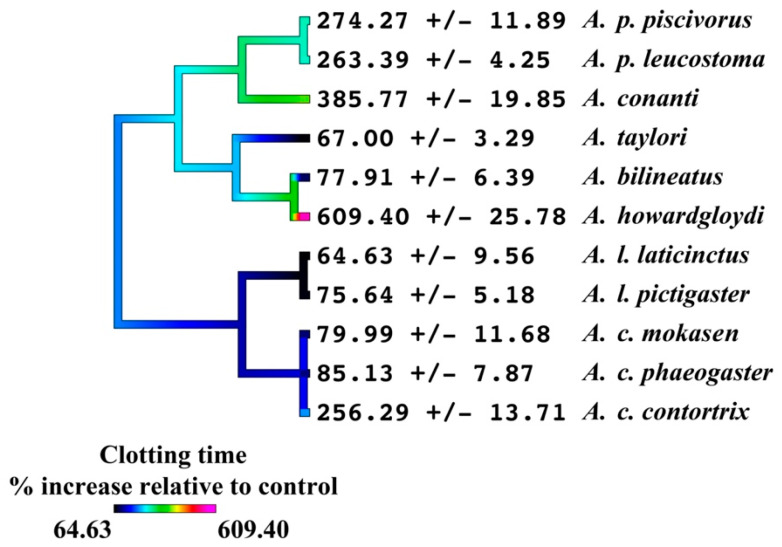
Ancestral reconstruction of elative inhibition of Factor Xa, showing the proportional increase in clotting time relative to the control (no venom effect = 0%). Phylogeny based upon Burbrink [2]. Data are n = 4 mean ± standard deviation.

The incubation tests of the activated enzymes Factors XIa, IXa, Xa, and IIa (also known as thrombin) revealed that FXIa was not significantly inhibited by any of the venoms; FIXa was significantly inhibited (*p* < 0.05) by *A. bilineatus*, *A. contortrix contortrix*, *A. contortrix mokasen*, *A. contortrix phaeogaster*, *A. howardgloydi*, and *A. laticinctus laticinctus*, with *A. howardgloydi* dramatically the most potent; FXa was significantly inhibited (*p* < 0.05) for all venoms, with *A. conanti*, *A. contortrix contortrix*, *A. conanti*, *A. howardgloydi*, *A. piscivorus leucostoma*, and *A. piscivorus piscivorous* the most potent; and FIIa (thrombin) was not significantly affected by any of the venoms (Figure 2 and Figure 3). As the taxa that most potently inhibited FXa do not form a monophyletic clade (Figure 4), this suggests that FXa inhibition is a basal trait within this genus that has been amplified on three occasions and therefore an indication of convergence in adaptation. This is consistent with FXa inhibition having been documented in other pit vipers from the Americas, including the *Bothriechis*, *Bothrops*, *Cerrophidion*, *Crotalus*, and *Ophryacus* species [21,22,65,66]; Asian pit vipers, including the *Deinagkistrodon* and *Gloydius* species [65,67,68]; true vipers, including the *Cerastes*, *Daboia*, *Pseudocerastes*, and *Vipera* species [65,69,70,71,72,73]; and elapid snakes including the *Naja* and *Pseudechis* species [14,41,74,75]. The toxin types responsible are diverse, including Kunitz peptides, lectin-framework toxins, and phospholipases A_2_.

To investigate the depletion of the phospholipid cofactor or zymogen degradation, plasma incubation was followed by the addition of an activated enzyme (Figure 2 and Figure 3). This revealed that the clotting cascade steps between that of FXIa and FIXa were affected by *A. bilineatus*, *A. conanti*, *A. contortrix contortrix*, *A. contortrix mokasen*, *A. contortrix phaeogaster*, and *A. howardgloydi;* that those of FIXa and FXa were affected by *A. bilineatus*, *A. contortrix mokasen*, *A. contortrix phaeogaster*, *A. howardgloydi*, and *A. piscivorus leucostoma;* and those of FXa and FIIa were affected by *A. contortrix mokasen*, *A. contortrix phaeogaster*, and *A. howardgloydi* (Figure 2 and Figure 3). The depletion of phospholipid levels by PLA_2_ toxins has been shown to inhibit the activity of FXa due to this enzyme’s requirement of phospholipids as binding cofactors [76]. Therefore, the lessened activity of FXIa and FIXa when added to plasma preincubated with venom may also be due to lowered phospholipid levels. In this case, there would be an equal distribution of effect for the FXIa, FXIa, and FXa additions after preincubation, a pattern seen for *A. bilineatus*, *A. contortrix mokasen*, *A. contortrix phaeogaster*, and *A. howardgloydi.* The most potent effects were noted for *A. contortrix mokasen* and *A. howardgloydi*, indicative of the convergent amplification of this trait. Therefore, for these species, the effects upon the activity of FXIa, FIXa, and FXa when added to the plasma preincubated with venom appear to be due to phospholipid degradation. However, for *A. conanti*, only the addition of FXIa was affected, not the downstream FIXa or FXa pathways. This therefore suggests that *A. conanti* degrades the FIX zymogen. Zymogen destruction has been previously described for pit vipers from the Americas, such as the destruction of the FX zymogen by *Crotalus mictlantecuhtli mictlantecuhtli* and *Crotalus mictlantecuhtli oaxacus* [51]. Future work, however, would be required to confirm the hypothesis that *A. conanti* venom destroys the FIX zymogen.

**Figure 5:**
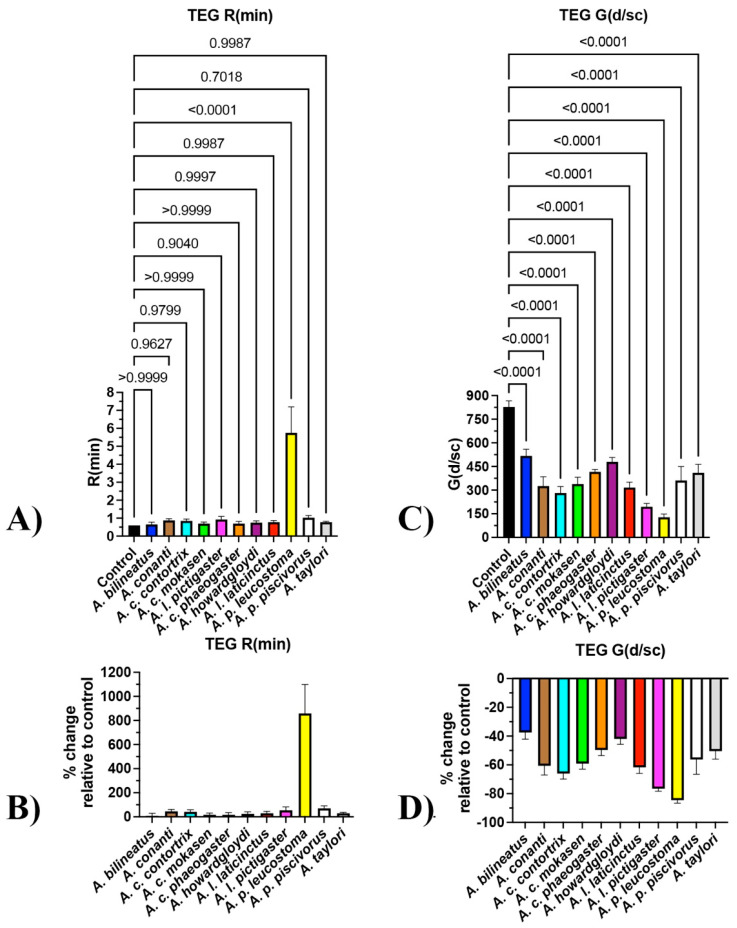
Thromboelastography tests where venom was incubated with fibrinogen without forming a clot, with thrombin subsequently added to form. (**A**) Thromboelastography R values (reaction time) representing the time taken from the start of the test until initial fibrin formation began. This measurement reflects the speed at which clotting starts. As the fibrinogen test was run under Claussian conditions, in which an excess of thrombin was added, any delay in R is reflective of depletion of fibrinogen levels. (**B**) R values as proportional increases in clotting time relative to the control (no venom effect = 0%). (**C**) Thromboelastography G values, which each represent the shear elastic modulus strength of a clot, which quantifies the clot’s firmness. The G value is expressed in dynes per square centimetre (d/sc) and provides a direct measure of the strength and stability of the blood clot formed during the test. Lower values indicate weaker clots. (**D**) G values as proportional increases in clotting time relative to the control (no venom effect = 0%; negative values indicate decreases in clot strength). *p*-values in (**A**,**C**) for each venom relative to control are from Brown–Forsythe and Welch ANOVA tests with post hoc Dunnett T3 multiple comparisons. Data are n = 4 mean ± standard deviation.

Thromboelastography was utilised to examine the direct effects upon fibrinogen (Figure 5). None of the venoms clotted fibrinogen in the pseudo-procoagulant manner seen in diverse pit vipers such as the *Crotalus*, *Gloydius*, and *Protobothrops* species [17,18,19,77] and true vipers such as the *Bitis* species [78]. Subsequently, we ascertained the effects upon fibrin clot formation and strength by incubating fibrinogen with the venoms and subsequently adding thrombin to trigger clot formation. *A. piscivorus leucostoma* was the only venom to delay clot formation (Figure 5A) relative to the control (Figure 5B). This is consistent with depletion of fibrinogen levels due to destructive cleavage. This is in contrast to the delay of the onset of clot formation and the diminishing of the speed of clot growth when whole plasma is used [79], which is due to contributory effects observed upon upstream clotting factors (Figure 2 and Figure 3). The direct degradation of fibrinogen is a trait that has been documented in the venoms of other pit vipers, such as the *Crotalus*, *Gloydius*, and *Protobothrops* species [17,19,20], and true vipers, such as the *Bitis* and *Causus* species [15,78]. However, clot strength was affected by all species (Figure 5C) relative to the control (Figure 5D). This is consistent with cleaving fibrinogen in a non-clotting manner but one that produced damaged fibrinogen still able to be cleaved by thrombin and form into a weak, unstable fibrin clot. Therefore, species that did not impede coagulation can still produce pathophysiological outcomes by damaging fibrinogen such that any clots formed (by prey or a human bite victim) would be weak and short-lived, resulting in an anticoagulative state of reduced or corrupted clotting potential, thereby promoting or facilitating pathologies such as internal bleeding and haemorrhagic shock.

**Figure 6:**
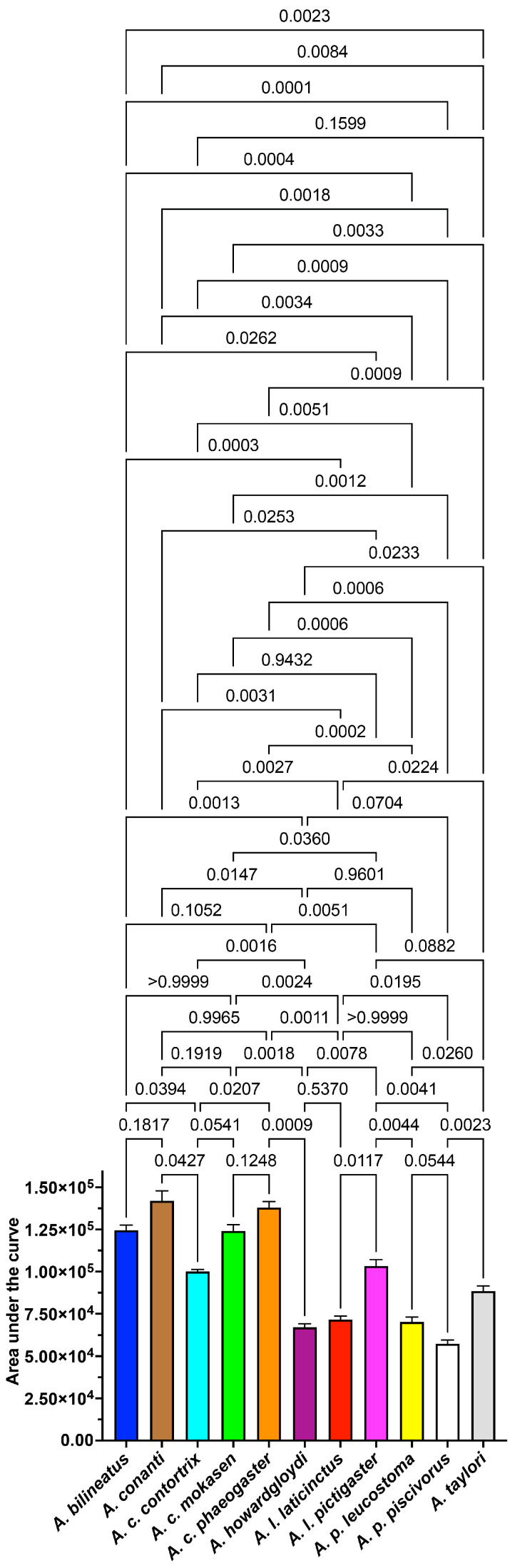
Relative activations of Protein C. *p*-values are from Brown–Forsythe and Welch ANOVA tests with post hoc Dunnett T3 multiple comparisons. Data are n = 4 mean ± standard deviation.

Subsequent assays were undertaken to ascertain the relative conversion of the Protein C zymogen into an activated form (Figure 6), as this is of interest not only in the context of venomous snake predatory ecology but also due to such venom enzymes having been of significant drug design and development interest, exemplified by their use in the development of the drug Protac^®^ isolated from *A. contortrix contortrix* [80]. Protein C, once activated to Activated Protein C (APC), inactivates clotting factors Va and VIIIa. This inactivation prevents the overproduction of thrombin and thus controls the formation of blood clots. Protein C also influences fibrinolysis (the process of breaking down clots) by modulating the activity of plasminogen activator inhibitor-1 (PAI-1), thereby promoting the breakdown of fibrin clots.

**Figure 7:**
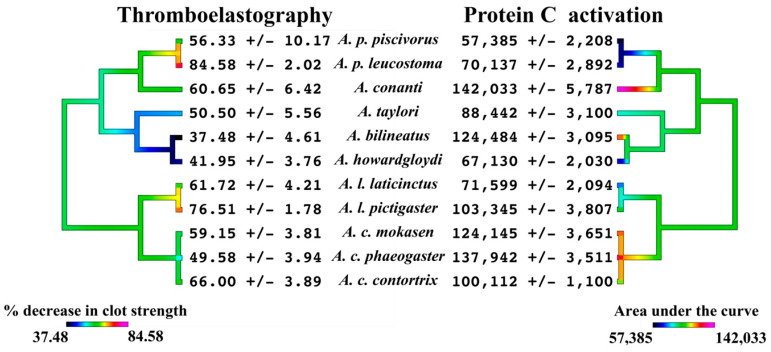
Ancestral reconstruction of the inverse relationships between the relative thromboelastographic determination of effects upon clot strength and the relative ability to activate Protein C. Phylogeny based upon Burbrink [2]. Data are n = 4 mean ± standard deviation.

Placed into a phylogenetic context (Figure 7), both the ability to directly damage fibrinogen, such that thrombin forms weaker clots, and the ability to activate the endogenous enzyme Protein C (which inactivates clotting factors Va and VIIIa and promotes fibrinolysis) are traits basal to the genus, but both traits have each been independently amplified on several occasions. The damaging action upon fibrinogen is at the most potentiated in the last common ancestor of the *A. laticinctus* taxa within the copperhead clade and the last common ancestor of the *A. piscivorus* taxa within the moccasin clade (Figure 7). For Protein C activation, this has been potentiated in the last common ancestor of the *A. contortrix* taxa while being amplified on two separate occasions within the moccasin clade, once in *A. bilineatus* and again in *A. conanti* (Figure 7). Conspicuously, the two actions appear to be negatively correlated but not entirely mutually exclusive, with the venoms amongst the most potent for one action being amongst the least potent for the other (Figure 5, Figure 6 and Figure 7).

The intraspecific and interspecific variability in *Agkistrodon* observed in this study regarding increases in clotting time, decreases in fibrinogen levels, and decreases in fibrin clot strength and prior proteomics data [3] are consistent with clinical reports of intraspecific and interspecific variability in increased PT and aPTT times, hypofibrinogenemia, and bleeding complications [4,64]. From a clinical perspective, these variations may reflect the differential ability to cause haemostatic disorders in the envenomed patient, which may impact diagnosis and management plans, including relative antivenom efficacy. The clinical implications of these findings are significant for understanding snakebite envenomation and its treatment, especially when considering the various nuanced angles of attack that can result in the same or similar coagulopathies. Clinical reports have described alterations in clotting parameters in a proportion of cases inflicted by *Agkistrodon* species [8,81]. Variability in venom effects can lead to different clinical presentations, such as increased prothrombin time (PT), activated partial thromboplastin time (aPTT), hypofibrinogenemia, and bleeding complications. Clinicians need to be aware of the potential variability in envenomation effects to accurately diagnose and manage snakebite cases. This may involve tailored therapeutic approaches and supportive care based on the specific venom profile of the snake involved. However, the variability in venom composition also underscores the need for broad-spectrum antivenoms that can neutralise a range of venom effects by reinforcing current viperid antivenoms with relevant *Agkistrodon* species so as to widen their neutralising scope.

From an evolutionary perspective, these variations may reflect adaptive significance, natural selection, and genetic drift and gene flow. The observed variability in the venom impacts upon clotting time, fibrinogen levels, and fibrin clot strength may reflect evolutionary adaptations to different ecological niches or prey types. Future work should investigate effects on prey plasma, as this has been shown to be an area of dynamic variation [52,82,83,84,85]. The differences in venom effects may be the result of natural selection, where certain venom profiles confer a survival advantage in specific environments. Variability in venom properties might also arise from genetic drift or gene flow between populations, contributing to the diversity observed in clotting time and fibrinogen levels.

From a biodiscovery perspective, these variations reflect the potential to yield novel therapeutics or biotechnological applications such as diagnostic uses. The unique properties of snake venom proteins and enzymes, such as those affecting clotting time and fibrinogen levels, can inspire the development of new drugs for coagulation disorders, cancer, and other diseases. Studying the variability in venom effects can provide insights into the biochemical pathways involved in haemostasis and how they can be modulated. This can lead to the discovery of new therapeutic targets. Venom components can also be harnessed for biotechnological applications, such as developing diagnostic tools or novel biomaterials. Understanding the diversity in venom effects can expand the potential applications of these bioactive molecules.

In conclusion, the observed variability in venom effects across different species and populations of Agkistrodon highlights the intricate interplay between evolutionary adaptation, clinical implications, and biodiscovery potential. Evolutionarily, these variations underscore the adaptive significance of venom composition, driven by natural selection and genetic dynamics such as drift and gene flow. Clinically, this variability necessitates a nuanced approach to diagnosing and managing snakebite envenomation, emphasising the importance of the development of broader-spectrum antivenoms. From a biodiscovery perspective, the unique biochemical properties of snake venoms hold immense promise for the development of novel therapeutics and biotechnological applications. By harnessing the diversity in venom effects, we can unlock new pathways for medical and scientific advancements, ultimately benefitting a wide range of fields from haemostasis to cancer treatment and beyond. As such, the coagulotoxic properties of snake venoms represent a fascinating area of research that bridges evolutionary biology, pharmacology, and medicine.

## 3. Materials and Methods

### 3.1. Venoms

*A. c. contortrix* venom was donated to author J.C. by César Olmos (Cullera, Valencia, Spain).

*A. c. conanti* (Liberty County, FL, USA), *A. c. mokasen* (Powell County, KY, USA), *A. laticinctus pictagaster* (Pecos County, TX, USA), *A. laticinctus laticinctus* (Tarrant County, TX, USA), and *A. p. leucostoma* (Harris County, TX, USA) venoms were collected from specimens maintained in the serpentarium of the National Natural Toxins Research centre at Texas A&M University—Kingsville, USA and collected by author E.S.

*A. c. phaeogaster* and *A. taylori* venoms were collected by author E.S. from specimens caught in Cole County, Missouri (USA), and Sota La Marina, Tamaulipas (Mexico), respectively.

*A. howardgloydi* venom was collected by authors B.L. and J.C. from specimens collected in the Costa Rican Province of Guanacaste and maintained in captivity in the serpentarium of the Instituto Clodomiro Picado (University of Costa Rica).

*A. bilineatus* (Mexico) and *A. p. piscivorus* (USA) venoms were purchased from Latoxan (Valence, France).

### 3.2. Coagulation Methods

Human plasma work was performed under University of Queensland Biosafety Approval #IBC134BSBS2015 and Human Ethics Approval #2016000256. The Australian Red Cross (44 Musk Street, Kelvin Grove, QLD 4059, Australia) supplied human platelet—poor plasma (3.2% citrated) under research approval #16-04QLD-10.

The anticoagulant methods were as follows:

- (Clotting enzyme + venom) + plasma
  ○ Undertaken to ascertain sites of actions upon particular enzymes.
  ○ Step 1: 50 µL of venom + 50 µL of 0.025 M calcium + 25 µL of OK buffer + 50 µL of PPL + 75 µL of human plasma.
  ○ Step 2: 120 s incubation at 37 °C.
  ○ Step 3: Addition of 25 µL of enzyme.
- (Venom + plasma) + clotting factor
  ○ Undertaken to ascertain sites of action upon zymogens downstream from a particular enzyme.
  ○ Step 1: 50 µL of venom + 50 µL of 0.025 M calcium + 25 µL of OK buffer + 50 µL of PPL + 75 µL of human plasma.
  ○ Step 2: 120 s incubation at 37 °C.
  ○ Step 3: Addition of 25 µL of enzyme.
- Enzymes used:
  ○ Factor IIa (thrombin), Stago cat. #00611.
  ○ Factor VIIa, 1.5 µg/mL, Haemonetics cat. #HCVIIA-0031.
  ○ Factor IXa, 15 μg/mL, Haemonetics cat. #HCXIA-0160.
  ○ Factor Xa, Stago kit; cat. # 00311.
  ○ Factor Xia, 15 µg/mL, Haemonetics cat. #HCXIA-0160.
  ○ Factor XII (activated with kaolin), 15 µg/mL, Haemonetics cat. #HCXII-0155.

Thromboelastography was undertaken using the TEG5000 platform to determine the venom effects upon the strength and elasticity of the fibrin clots. The methods were as follows:

- Seven µL of venom was added to 72 µL of CaCl_2_, 72 µL of phospholipid, and 20 µL of Owren–Keller buffer, followed by 189 µL of fibrinogen and then run immediately for 30 min measurement of clot formation.
- As the venoms did not directly clot the fibrinogen, assays were completed with an additional step by adding 7 µL of thrombin followed by a further 30 min to determine if the fibrinogen had been destroyed by the venom to prevent clotting.

Protein C zymogen activation was undertaken using a Fluoroskan Ascent™ (Thermo Scientific, Vantaa, Finland) as follows:

- In total, 10 μL of phospholipid, 10 μL of venom (containing 1 μg of venom), and 10 μL of Protein C zymogen (containing 10 μg total of zymogen) were manually pipetted, followed by the automated pipetting of 70 μL of a mixture of buffer (5 mM CaCl_2_, 150 mM NaCl, and 50 mM Tris-HCl at pH 7.3) and fluorogenic peptide substrate (ES011 substrate Boc-Val-Pro-Arg-AMC; Boc: t-Butyloxycarbonyl; 7-Amino-4- methyl coumarin) into each experimental well of a 384-well plate.
- Activated factors were used in place of zymogens as positive controls.
- The zymogens were also replaced in the venom control wells by 10 μL of Fluoroskan buffer to determine the activity of the venom directly upon the substrate.
- Fluorescence generated by the cleaving of the substrate was automatically recorded by the machine. The results were obtained by subtracting blank values from reactions, followed by the subtraction of the venom without zymogen from the venom with zymogen.

### 3.3. Phytools Methods

Ancestral state reconstruction was undertaken in R-studio (Version 2024.04.2+764) using the following script:

library(ape)
library(maps)
library(phytools)
data<-read.csv(file.choose())
dat<-data
mapvar<-dat$var
names(mapvar)<-dat$species
tree<-read.tree(file.choose())
asr<-contMap(tree,mapvar,plot=F)
plot(setMap(asr,col=c(1,4,5,3,7,2,6)),lwd=10)

### 3.4. Statistical Analyses

Brown–Forsythe and Welch ANOVA tests with post hoc Dunnett T3 multiple comparisons were undertaken in Graphpad Prism 8.1.1 (GraphPad Prism Inc., La Jolla, CA, USA) to ascertain the statistical significance of the venom effects relative to the control values.

## Data Availability

All data are presented in the figures.
